# Exploring the Concept of In Vivo Guided Tissue Engineering by a Single-Stage Surgical Procedure in a Rodent Model

**DOI:** 10.3390/ijms232012703

**Published:** 2022-10-21

**Authors:** Clara Ibel Chamorro, Said Zeiai, Nikolai Juul, Oliver Willacy, Jinxing Huo, Jöns Hilborn, Magdalena Fossum

**Affiliations:** 1 Department of Women’s and Children’s Health, Bioclinicum J10:20 Karolinska Institutet, 171 76 Stockholm, Sweden; 2 Laboratory of Tissue Engineering Rigshospitalet, Faculty of Health and Medical Sciences, University of Copenhagen, 2100 Copenhagen, Denmark; 3 Department of Pediatric Surgery, Copenhagen University Hospital, Rigshospitalet, 2200 Copenhagen, Denmark; 4 Department of Material Science and Engineering, Uppsala University, 751 23 Uppsala, Sweden; 5 Department Chemistry Ångström Laboratory, Uppsala University, 751 20 Uppsala, Sweden

**Keywords:** autologous, tissue engineering, tensile strength, polymers, plastic compression

## Abstract

In severe malformations with a lack of native tissues, treatment options are limited. We aimed at expanding tissue in vivo using the body as a bioreactor and developing a sustainable single-staged procedure for autologous tissue reconstruction in malformation surgery. Autologous micro-epithelium from skin was integrated with plastically compressed collagen and a degradable knitted fabric mesh. Sixty-three scaffolds were implanted in nine rats for histological and mechanical analyses, up to 4 weeks after transplantation. Tissue integration, cell expansion, proliferation, inflammation, strength, and elasticity were evaluated over time in vivo and validated in vitro in a bladder wound healing model. After 5 days in vivo, we observed keratinocyte proliferation on top of the transplant, remodeling of the collagen, and neovascularization within the transplant. At 4 weeks, all transplants were fully integrated with the surrounding tissue. Tensile strength and elasticity were retained during the whole study period. In the in vitro models, a multilayered epithelium covered the defect after 4 weeks. Autologous micro-epithelial transplants allowed for cell expansion and reorganization in vivo without conventional pre-operative in vitro cell propagation. The method was easy to perform and did not require handling outside the operating theater.

## 1. Introduction

Reconstructive surgery is sometimes required after mutilating traumas, surgical treatments of cancer, congenital malformations, or other diseases where the patient’s native tissues are deficient. In cases with a severe lack of native tissues, substitution is sometimes difficult and treatment options are limited. Tissue engineering has therefore arisen as a possible solution for these patients [1,2].

Tissue engineering techniques have already been tested in clinical trials for substituting parts of organs, such as skin, bladder, arteries, and cartilage, but the clinical outcomes have so far been variable [1,2,3,4]. Some of the most successful results have been demonstrated with lethal burn injuries, where patients benefited from autologous skin grafts [5]. However, follow-up studies have suggested that cultured autologous skin may be fragile and cannot withstand multiple traumas [3].

For other clinical applications, such as for bladder augmentation, an initial study was described as successful, but a subsequent multicenter clinical trial needed to be discontinued due to adverse side effects, such as bladder rupture, probably due to decreased resistance to mechanical trauma [2].

Cartilage tissue engineering has also been explored in clinical trials, where the regenerative potential of mesenchymal stem cells was analyzed in different settings [6]. Post-transplantation analyses revealed quality issues concerning cell regeneration, mechanical properties, and integration of the scaffolds [7]. In analogy, engineering an arterial vascular graft with characteristics that meet the needs of high mechanical strength, high compliance, and a suitable rate of degradation, has been challenging [4].

Within the field of tissue engineering with autologous human cells, certain key steps are usually needed for in vitro expansion. Firstly, cells are harvested from the patient and expanded in a laboratory that needs to be certified for good manufacturing practice to procure safety and traceability [8]. Autologous cell harvesting can commonly be achieved mechanically by tissue biopsy, but, if not available, mesenchymal stem cells could be an alternative for expansion and differentiation into different lineages [9,10,11]. Secondly, the cells are seeded onto a scaffold that resembles the desired organ. So far, these scaffolds have commonly been two-dimensional flat sheet constructs [1]. Finally, the cells need to propagate on the scaffold before the autologous transplant is ready for surgical incorporation into the targeted organ [1,12,13].

At present, one of the main challenges of the technique is to provide a scaffold with suitable mechanical characteristics for the intended organ [1,4,14,15]. Based on prior studies with urothelial-tissue-engineered implants, a broad consensus has been established that acellular grafts produce inferior regenerative outcomes, although this hypothesis remains to be further challenged [16,17,18].

Altogether, the standard procedures for tissue engineering are time-consuming, costly, and require specialized laboratories, with highly trained personnel, and repeated surgical interventions (one for harvesting and one for reconstruction) [1,2].

To address this issue, we previously introduced a strategy aiming at expanding autologous tissues inside the body itself, instead of in the laboratory [19]. The method, referred to as plastic compression (PC), renders the transplant suitable for surgical handling by expelling water from a collagen gel that is incorporated into the scaffold and the autologous tissue [15,20,21]. This three-dimensional (3D) construct has previously been tested with expanded minced urothelium on top of a scaffold in vitro [19], and enabled cell propagation and reorganization within the transplant. One of the major advantages of this technique is the ability to expand small-sized excised tissue fragments to a relatively larger scaffold area (i.e., expansion ratio) after fragmentation.

As a next step, the strategy of this study was to use an in vivo model to evaluate whether the body itself could be used as a bioreactor for tissue expansion, and the scaffold as a 3D guide for the regenerated tissue and for achieving mechanical stability. Skin is easily accessible and involves less trauma to the animal than obtaining bladder mucosa. Preparatory in vitro experiments were performed to procure methodological relevance in rodent and porcine models before in vivo studies, which is in line with the 3R standards (replacement, reduction, and refinement).

The primary aims of this in vivo study were to evaluate tissue integration and biocompatibility of the composite three-dimensional plastic compressed scaffolds (3D PC scaffold) with autologous micro-epithelial transplants, and to compare these with similar acellular scaffolds. Secondly, we aimed to evaluate the tensile strength of the 3D PC scaffolds after implantation in vivo. Both aims were identified as major factors for reaching our ultimate objective; to achieve autologous in vivo tissue expansion and reconstruction in a single-stage surgical procedure.

## 2. Results

### 2.1. Minced Rodent Skin In Vitro

**Figure 1:**
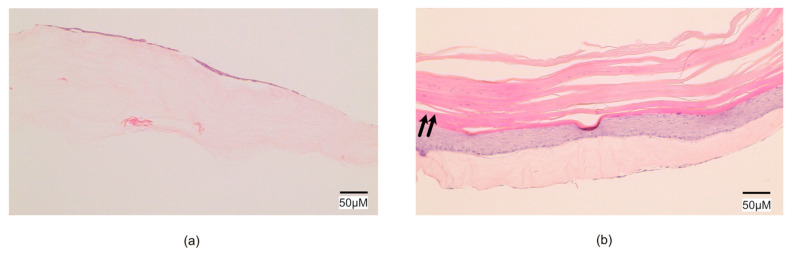
Epithelium covering the scaffold in vitro. Microphotograph of the transplants described in 2.1 with conventional H&E staining. (**a**) After 2 weeks of in vitro culture, a single-layer epithelium was present on top of the collagen. (**b**). After 6 weeks, a keratinized stratified (arrows) squamous epithelium was covering the collagen.

To assess the in vitro compatibility of the 3D PC constructs with minced tissue, we used minced rat skin and placed it at the top of the PC collagen in a 1:6 expansion ratio, as indicated in the materials and method section. The in vitro cultures of the 3D PC constructs showed no signs of delamination between the knitted fabric and the collagen. H&E stains demonstrated a continuous single-cell layer of epithelium after 2 weeks (Figure 1a), and a multilayered keratinized epithelium was present after 6 weeks in vitro (Figure 1b), indicating cell migration from the minced particles towards the free surface of the 3D PC construct.

### 2.2. Porcine Bladder Wound Healing Ex Vivo

**Figure 2:**
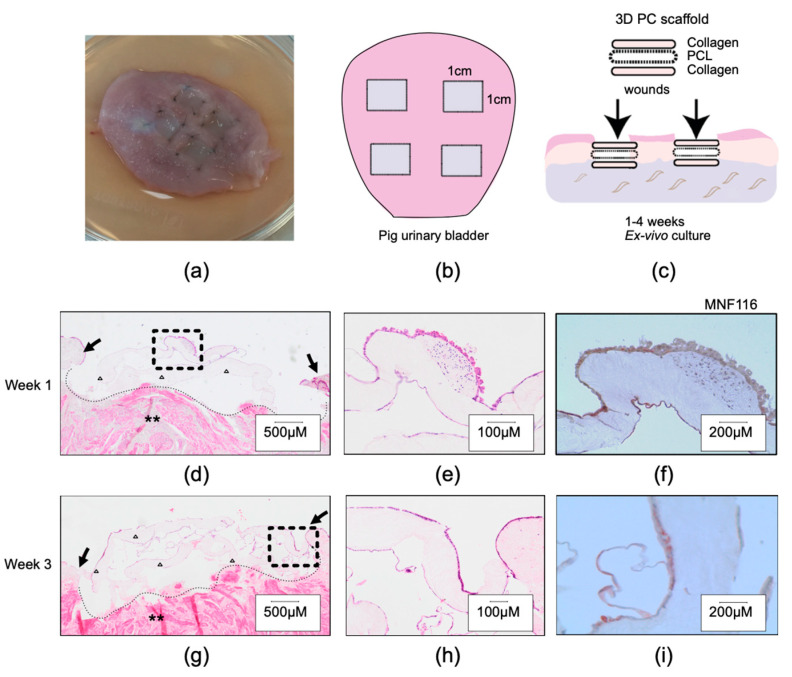
Ex vivo pig bladder wound healing setup and micromorphological findings. (**a**) Left upper panel showing the pig bladder with sutured implants to fill the partial thickness wounds. (**b**,**c**) Schematic drawing to illustrate the composition of the 3D PC implants and how these were positioned in the bladder. (**d**–**i**) Histological sections after 1 week in culture (upper panel) and after 3 weeks in culture (lower panel), stained with H&E (**d**–**e**,**g**,**h**) and cytokeratin (**f**,**i**). Note: arrows indicate the wound margins, native bladder submucosa (asterisk), and the 3D PC scaffold on top of the ex vivo wound (triangles **e**,**f**,**h**,**i**). Region of interest magnified from (**d**) and (**g**), respectively.

To test the concept of tissue repair using 3D PC scaffolds, we next used an ex vivo organ model for bladder wound healing. In this model, the whole urinary organ was collected, and four ex vivo partial wounds were created. Histological evaluations of the ex vivo model showed increased wound cellularity during the first and third weeks of healing (Figure 2). These cells expressed epithelial cell markers (MNF116) (Figure 2f,i) and a urothelial cell morphology (Figure 2e,h). At the third week, all surfaces were covered by a neo-urothelium of two to three cell layers (Figure 2g–i).

### 2.3. In Vivo Results

To assess the in vivo compatibility of the 3D PC construct with and without minced skin tissue, we next used a rat subcutaneous implantation model and analyzed the local inflammatory and wound healing responses.

**Figure 3:**
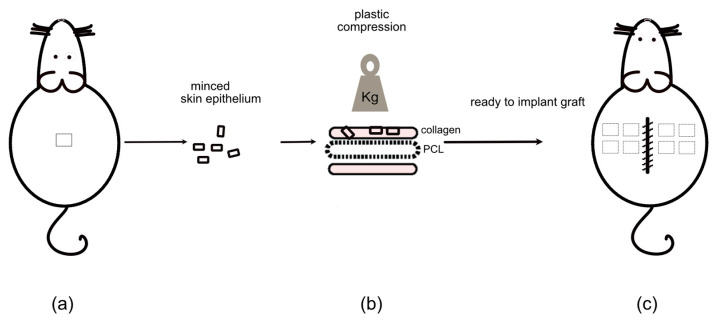
Diagram of the in vivo model. (**a**,**c**) Schematic illustrating the position of the 1 × 1 cm biopsy on the back of the rat and the subcutaneous placement of the scaffolds (with or without minced tissue transplants). (**b**) Illustration of how the skin biopsy was minced, combined with collagen and PCL, and prepared by plastic compression before grafting back to the same rat in the subcutaneous space of the back.

**Figure 4:**
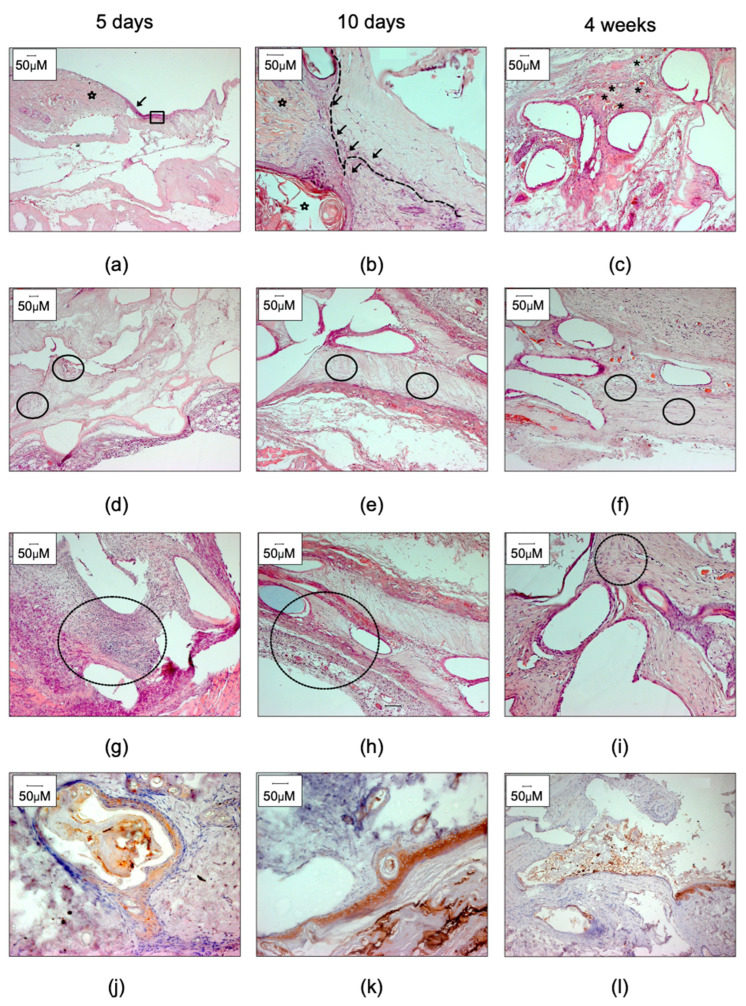
Histological images demonstrating the interactions between the 3D PC constructs and surrounding native tissue at different time points. (**a**) H&E staining at day 5, epithelial cells (arrow) were observed from the edge of the minced tissue (star) to the collagen parts of the scaffolds (square). (**b**) Neo-epithelialization was also observed 10 days after transplantation, and single-layer keratinocytes were visualized in clusters near the minced tissues (arrows and dotted line). (**c**) At 4 weeks, minced particles were hard to distinguish from the surrounding tissue, and regions with high content of new vessels were identified (asterisk). (**d**–**f**) Fibroblast-like cells and inflammatory cells (circles) were identified within the collagen parts of the 3D PCscaffolds. (**g**–**i**) The presence of inflammatory cells decreased with time (circles) in the 3D PC scaffolds. (**j**–**l**) Stratified squamous epithelium originated from grafted minced particles. H&E (**a**–**i**) and pan-cytokeratin immunostaining (**j**–**l**).

All rats survived the course of the study period. The subjects did not demonstrate any complications and seemed unaffected by the implants. All implants were found at the site of the initial transplantation (Figure 3). Evaluation of cell ingrowth, visible capillaries, and loosening and thinning of collagen was used to reveal integration of the 3D PC scaffolds (Supplementary Figure S1). At 5 days, there were zones of neo-epithelium adjacent to the minced skin in the 3D scaffold (Figure 4a, dashed line and arrow). A multilayered epithelium, resembling keratinized stratified squamous epithelium, was observed at 10 days (dashed line and arrow in b) and immunohistochemistry performed in samples at every time point confirmed its skin keratinocyte origin (Figure 4j–l). None of the controls (in 4d–i) presented any epithelial growth and cells could be observed inside the collagen part of the transplant in both controls (Figure 4d–f; dashed circles) and 3D PC scaffolds with minced skin (supplementary Figure S2d,h,l). After 4 weeks, the collagen was completely integrated (cellularized) in most areas of both control (Figure 4c,f,i and supplementary Figure S2i,j) and 3d PC scaffolds with minced skin (supplementary Figure S2k,l). Fibers from the knitted fabric were observed intact during the whole study period in most of the samples (supplementary Figure S2, asterisk). All transplants, with and without minced skin, produced inflammatory cell infiltration (Figure 4g–i for controls and supplementary Figure S2a,b for 3d PCLscaffolds with minced skin). After 4 weeks, inflammatory cell infiltration was reduced but was still present (Figure 4i, circles marking zones with inflammatory cells) and supplementary Figure S2k–l).

**Figure 5:**
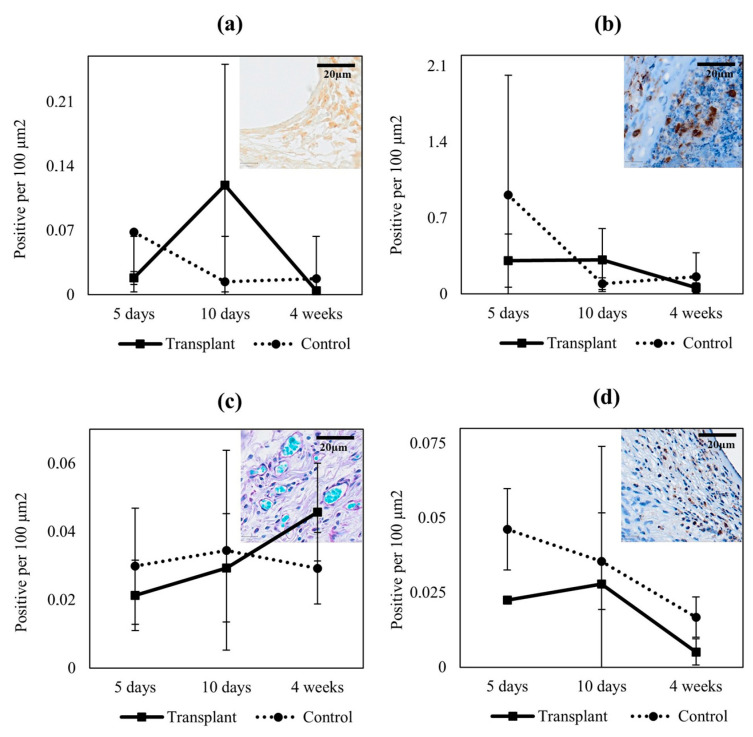
Quantification of proliferation, inflammation, and vascularization. *(***a**) Proliferative cells were identified and counted in ki67 staining. (**b**) Macrophages were identified with CD68. (**c**) Vascularization was evaluated in PAS staining. (**d**) Inflammation was evaluated with neutrophil myeloperoxidase staining for early phagocytosis.

Quantitative analysis of the proliferative marker showed increased numbers of proliferative cells after 10 days in the transplants with minced tissue compared to controls; however, the difference was not statistically significant (Figure 5a). Quantification of inflammatory markers displayed no significant difference in either early nor late inflammation between transplants with or without minced tissue (Figure 5b,d). Histological quantification of tissue vascularization revealed increased numbers of vascular structures after 4 weeks in the transplants with minced tissue compared to controls, although not statistically significant (Figure 5c). 

### 2.4. Tensile Strength

**Figure 6:**
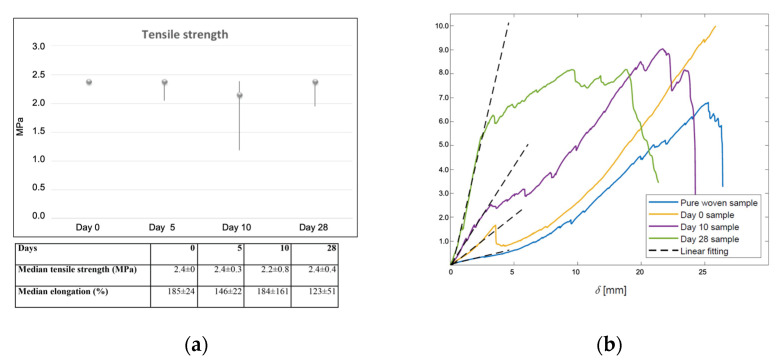
Tensile strength and elongation of transplants. (**a**) Measured tensile strength (median and variation) of 3D PC scaffolds, at different integration time points are plotted in the diagram (maximum measurable strength was 2.4 MPa). Corresponding median strength and relative elongation at different time points in the table below. (**b**) Elasticity demonstrated as load-displacement curves from tensile tests and the corresponding linear curve fitting results. The stiffness, indicated by the inclination of the fitted dash lines, were 0.13 N/mm (control), 0.42 N/mm (0 days), 0.83 N/mm (10 days) and 2.30 N/mm (28 days).

The implants kept their tensile strength throughout the experiment. The highest possible measured strength was 2.4 MPa and most transplants were intact at this point. The tensile strength declined, from a median of 2.4 MPa at the start of the experiment, to a median of 2.2 MPa after 10 days with higher variability. However, after 4 weeks, the transplants had regained their former strength of 2.4 MPa (Figure 6a). The elasticity of the construct decreased over time. The elongation, or elongation at break, was unchanged from a median of 185% at the start of the experiment to 184% at 10 days, but was reduced to 123% after 4 weeks (Figure 6b).

## 3. Discussion

We aimed to improve our method for autologous tissue grafting, and examined in vivo qualities such as tolerability, tissue integration, and mechanical properties of the transplants that we previously had only studied in vitro [22,23]. To do this, we used an in vivo small animal model.

All in vivo transplants were tolerated by and incorporated into the adjacent tissues, and we successfully demonstrated cell proliferation in vivo. We did not find any negative side effects, such as expulsion of the transplant, or inconveniences demonstrated by the rats.

Histologically, we found that the scaffolds were well-integrated after 10 days, as demonstrated by vascularization within the construct, and totally integrated with the surrounding tissue after 4 weeks, as indicated by the overall histology. We found comparable levels of inflammation in the transplants with and without autologous tissue, and tendencies of increased proliferation and angiogenesis in transplants with minced tissue; however, the study was not designed for comparing cell regeneration capacity between tissue-loaded transplants and controls. On top of the constructs, epithelial cells were observed after 5 days, which indicated migration and proliferation of cells from the transplanted minced tissue in the collagen, as well as reorganization to the surface of the scaffold. A mature keratinized stratified squamous epithelium was present after 10 days.

We previously identified that the scaffold needs to possess certain properties, such as mechanical strength, to sustain surgical handling and manipulation. It also needs to keep its strength over time, to allow take of transplants, and to sustain its characteristics to fulfill future demands related to the organ of interest (i.e., bladder, intestine, or skin) [15,19]. We therefore explored the mechanical properties of the transplanted biomaterial in vivo after implantation. We used a device that measured the elongation values in a standardized manner and within the scale of normal bladder tissue. The 3D PC scaffold kept its tensile strength during the whole experiment, and conserved most of its elasticity despite the static conditions prevailing in the present experimental model. Our measurements demonstrated tensile strengths up to 2.4 MPa, which is within the order of magnitude for normal human bladders [24]. These qualities could make the scaffold suitable for tissue engineering of organs in need of elasticity, such as the urinary tract. However, to elucidate this, additional studies including dynamic changes would be needed, most likely in a larger animal model.

The elasticity of the scaffolds was also investigated. Since the scaffolds were discrete structures, especially the non-implanted controls, the elastic modulus could not be used; instead, a corresponding parameter, stiffness, was used to quantify the elasticity. The force–displacement curves obtained from the tensile strength tests were post-processed. The stiffness of the scaffolds, as indicated by the inclination of the initial elastic part of the curve, was determined through linear curve fittings, as shown in Figure 6. As seen, the initial parts of all the curves are close to linear, indicating a linear deformation in the initial stage of the sample stretching. We observed a monotonic increase in stiffness with integration time in vivo. This can be explained by improved tissue integration that contributed to the biomechanical tolerance. When the integrated tissue broke at a certain elongation, the stiffness dropped to become similar to the control samples.

From previous studies, we knew that our composite scaffold was favorable for urothelial proliferation, both with cultured porcine cells, seeded from cell suspension after conventional in vitro culture, and with minced tissue particles [21]. We therefore aimed to evaluate the scaffold with minced rodent skin in vitro to make sure our scaffold was also feasible in this model. This was performed before initiating our in vivo studies. In these studies, we found that a single epithelial layer had formed after 1 week, and a keratinized stratified squamous epithelium appeared after 6 weeks in vitro; the study design was therefore deemed adequate.

The rationale for using a small animal model was to evaluate the composite biomaterial, including minced autologous epithelium, after in vivo transplantation. At this step, where we only wanted to study tissue integration and epithelial tissue expansion, we used the rodent skin epithelium model as a proof-of-principle in line with animal ethical principles [25]. However, based on the abnormal subcutaneous position of the transplanted epithelium, normal skin expansion and differentiation was not expected.

Other researchers have studied how human cells in single suspension (fibroblasts or muscle cells) can be integrated into collagen before plastic compression and cell culturing for cell expansion [26,27,28]. To our knowledge, no other groups have performed minced tissue integration into PC collagen, circumvented enzymatic manipulations and in vitro culturing of autologous cells, nor conducted in vivo studies on PC transplants.

Our goals for bladder augmentation would need to be performed in a large animal model. At this point, we did not intend to incorporate the scaffold into the urinary bladder of the rat due to its small size. When evaluating a transplant, with respect to integration and regeneration, it is crucial to evaluate a critical size defect that cannot be healed solely by contraction or ingrowth from the borders. A small-sized bladder defect would therefore not have given results representative of human bladders. Therefore, as a first step, we evaluated the basic characteristics of the scaffolds, such as integration, tolerance, and tensile strength, over time.

To evaluate whether our composite scaffold could be sutured to the porcine bladder and eventually integrated into the bladder wall, we used a simplified wound healing model with porcine bladder tissue ex vivo. In this model, partial thickness wounds were created on the submucosal layer of the bladder wall. Autologous minced urothelial transplants were thereafter inserted into the missing areas of epithelium. We found that cells migrated and proliferated into the composite scaffold to cover the wound, forming a single-cell layer within a few days. All wounds healed with a multilayered neo-urothelium over the scaffold. This further demonstrates a favorable healing capacity in a porcine setting, which strengthens our present initiatives and future goal to develop a bladder augmentation transplant.

Weaknesses of this study include the non-physiological placement of subcutaneous transplants with skin epithelium in the in vivo study. In our initial pilot study, we used a rodent full-thickness skin wound model for the experiments, and by these means, the scaffolds were placed at the bottom of full-thickness skin wounds that were kept moist and covered with dressings, as described in studies by other authors [29]. We soon noticed that the rats could escape their dressings and damage the transplants. We also noticed that the contraction of the rat skin was pronounced, and would disturb further evaluation of the neo-regeneration around the transplant itself. Despite applying methods as described in previous studies, we could not prevent these contractions [30]. Instead, we decided to evaluate the transplants subcutaneously. In this model, the rats could not influence the transplant by scratching or biting, and better evaluation of scaffold properties could be reassured. In addition, the subcutaneous implantation did not seem to disturb the rats. The downside was that epithelial cells could not expand to cover and reorganize over the whole scaffold surface, most likely due to a lack of air exposure that would have guided proliferation and reorganization. Therefore, as expected, the epithelial cells grew in clusters or in smaller areas on top of the collagen.

We confirmed that it is possible to expand minced epithelium in vivo by placement on top of the plastically compressed scaffold. In addition, we found that the procedures were safe for the animals, as indicated by the absence of graft versus host reactions, and a decrease in inflammation over time. Our findings indicate that after 4 weeks, the constructs were integrated inside the body, with or without minced tissue, during the healing process. With this information, we can plan future in vivo studies aimed at integrating the scaffold into the urinary system in a large animal model where a critical size defect can be established and reconstructed with the 3D PC scaffold.

## 4. Materials and Methods

### 4.1. Cell Culture Media

Cell culture media was prepared with a 4:1 mix of DMEM:Ham’s F12, (Gibco-BRL Life Technnologies Europa, Bleiswijk, The Netherlands) and supplemented with fetal bovine serum 10% (Life Technnologies Europe, Bleiswijk, The Netherlands ), insulin 5 µg/mL, hydrocortisone 0.4 µg/mL, adenine 21 mg/mL, cholera toxin 10^−10^ mol/L, triiodothyronine 2 × 10^−9^ mol/L, transferrin 5 µg/mL, epidermal growth factor 10 ng/mL (all from Sigma-Aldrich, St. Louis, MO, USA), penicillin 50 U/mL, and streptomycin 50 µg/mL (Invitrogen).

### 4.2. Knitted Polymer Fabric

Fabrication of the degradable knitted mesh of poly (ɛ-caprolactone) and pre-activation of the surface was performed as previously described, to optimize its integration into collagen fibers [21]. In short, poly(ɛ-caprolactone) (Sigma-Aldrich St. Louis, MO, USA), with an average molecular weight of 80,000 g/mol, was dried in vacuum and compressed into cylindrical rods before melt spinning at 180 °C in nitrogen atmosphere. The knitting was performed with an 89 mm diameter cylinder with 380 needles under an air pressure of 40 psi (3 bar). Pre-activation of the polymer surface was completed by placing the knitted fabric in 2.5 M NaOH for 40 min and subsequent treatment in PVA solution (Merck Stockholm, Sweden; 72,000 g/mol, 1% *w*/*v*) for 10 min.

### 4.3. Tridimensional Scaffold Construction including Plastic Compression

The 3D PC scaffolds were constructed from two layers of collagen and one middle layer of knitted polycaprolactone (PCL) fabric as described above [21]. In short, eight parts of sterile rat tail collagen type I was mixed with one part of 10X Dulbecco’s modified Eagle Medium (DMEM) (Gibco-BRL Life Technnologies Europe, Bleiswijk, The Netherlands ). After the pH had been neutralized with 2.5 M NaOH, one part of 1X DMEM (Gibco-BRL Life Technnologies Europe, Bleiswijk, The Netherlands ) was added. A rectangular sterile stainless steel mold (1 × 2 × 3 cm in height x length x width) was filled with 3 mL of the solution and incubated for 10 min at 37 °C. One sheet of the PCL knitted fabric was placed on top of the collagen hydrogel and, subsequently, 3 mL of the mold was filled with collagen solution. Gel formation was achieved in an incubator after 20 min. Thereafter, the minced tissue (skin particles) was distributed on top of the scaffold. The construct was placed on a nylon mesh (closest to the collagen hydrogel), a stainless steel mesh, and gauze pads. The gel was covered with a nylon and a steel mesh before plastic compression. Removal of excess water by PC was performed with a static loading plate of 120 g on top of the assembly for 5 min at room temperature, to create the autologous micro-epithelial transplants [31].

### 4.4. In Vitro Experiment with Rodent Skin

First, 1 cm^2^ of epithelium (1 × 1 cm) from the back part of the rats was collected and minced using a mincing device (Xpansion^®^ micrografting handheld device, Applied Tissue Technologies, Hingham, MA, USA). The minced skin was then distributed on top of 6 cm^2^ collagen–PCL–collagen in a 1:6 expansion rate (1 cm^2^ minced skin to 6 cm^2^ scaffold surface) before plastic compression to obtain the 3D PC scaffold, as described in the section above. The scaffold was divided into six pieces of equal size and cultured in 12-well plates in cell culture medium. The wells were kept in an incubator with 5% carbon dioxide and humidified air at 37 °C at atmospheric pressure. The medium was changed every second day. After 2, 4, and 6 weeks, respectively, two samples were collected and fixed in 4% formaldehyde.

### 4.5. In Vitro Experiment with Porcine Bladder

To test the concept of tissue repair using 3D PC scaffolds, we next used an ex vivo organ model for bladder wound healing. Porcine urinary bladders were collected directly postmortem, after interventions and purposes not related to the study, under sterile conditions, and transported to the laboratory. Once in the laboratory, the bladders were opened and four 1 cm^2^ partial thickness wounds were created by removing the mucosa layer and most submucosa. The 3D PC scaffolds were sutured within the submucosa to fill the wounded area (1 cm^2^ squares), using four Ethilon 5-0 (Ethicon, Sommerville, NJ, USA) stitches, at each corner of the transplants (a visual description can be found in Figure 2. The bladder wounds were thereafter placed in culture dishes with freshly prepared cell culture medium and kept in an incubator with 5% CO_2_, at 37 °C. The medium was changed every day during the first week, and then every other day. The closure of the wound was observed macroscopically, and samples were collected and fixed after 1, 2, and 3 weeks in culture.

### 4.6. In Vivo Experiments

Twelve male rats (Sprague Dawley) were put in general anesthesia with sevoflurane (Virbac, Carros, France) and then placed on the operating table with maintained inhalation of sevoflurane using a mask during the whole procedure.

The back of the rat skin was waxed with Veet Easy Wax Removal Strips (Reckitt Benckiser, Slough England, UK) and sterilized with Sterillium (Hartmann, Heidenheim, Germany). A 1 cm^2^ full skin biopsy was performed on the skin, and the subcutaneous tissue was removed mechanically with scissors, leaving only the upper dermis and epidermis (approximately 0.8 mm thick). A mincing device (Xpansion^®^) was used to cut the upper dermis and epidermis into smaller pieces, horizontally and then vertically, to approximately 0.8 × 0.8 mm pieces. The autologous minced tissue was prepared at the same time as the 3D PC scaffolds. Minced skin tissue was distributed on top of the scaffold in a 1:6 expansion rate and controls were generated without tissue transplants. In nine of the rats, evenly distributed 3D PC scaffolds with or without minced tissue transplants were sutured subcutaneously on the left or right side of the back with four anchoring Ethilon 4-0 stitches (Ethicon, Sommerville, NJ, USA) (Table 1). Eight samples were transplanted per rat (four with and four without autologous tissue). For each piece with tissue transplants on top, one control without minced tissue was sutured on the contralateral side of the back. The distribution between right and left side was randomly chosen between tissue-loaded 3D PC scaffold and control. The wounds were closed with Ethilon 4-0 running sutures (see Figure 3). A bandage was placed on the wound and the rats were observed until fully awake. All subjects were kept together in groups of two to three rats. After 5 days, 10 days, and 4 weeks, respectively, the rats were anesthetized, the samples surgically removed, and finally, the rats were terminated. In the three remaining rats, acellular 3D PC scaffold implantations were used for tensile strength analyses at the same time points (described further below).

A total of 12 rats were operated on, with the insertion of a total of 36 implants containing minced tissue and 51 implants without. A total of 72 samples from the three time points (5, 10, and 28 days) were collected for histological analysis and 15 samples were collected for tensile strength measurements (Table 1).

### 4.7. Histological Evaluations

All tissue samples collected at the end of each time point were fixed in buffered 4% formaldehyde, dehydrated, and embedded in paraffin before cutting into 5 μm sections. Rehydration was performed before staining with hematoxylin–eosin (H&E) for routine histology. Qualitative and quantitative histological evaluations were performed with respect to tissue and collagen integration, neovascularization, and inflammatory response.

Immunostaining was performed as previously described [32] using specific antibodies against pan-cytokeratin (MNF116), proliferative marker ki67, and the inflammatory markers myeloperoxidase (for neutrophil detection) and CD68 (macrophage marker) (Table 2). 

PAS staining was performed following the manufacturer’s instructions (Abcam) for capillary quantification. The vessels were identified by the presence of a well-defined lumen containing erythrocytes, and each independent luminal structure was included although adjacent to another. 

Each slide was scanned using an Olympus VS200r esearch slide scanner (Olympus Life Science corporation, Tokio Japan) at the Histocore facility located at Bioclinicum, Karolinska Institutet).

All specimens were independently reviewed in toto by two authors (NJ, OW), blinded to time points and study conditions (i.e., transplant without or without autologous tissue). Using the QuPath software for digital pathology analysis (developed by the University of Edinburgh), the complete transplant area below the subcutaneous shivering muscle was quantified either by manual counting or by automated cell detection (Supplementary Figure S1).

### 4.8. Tensile Strength

Fifteen 1.5 × 1 cm^2^ implants without minced tissue were implanted subcutaneously on the back of three different rats. The implanted samples, obtained from the in vivo experiment, were stored in formalin solution for protection during transportation before mechanical testing. As a control, we used the pure knitted polymer fabric (PCL) that had not been previously implanted. All were mechanically cleaned from excessive surrounding smooth tissue before performing measurements. The samples were rinsed in distilled water and rehydrated in phosphate-buffered saline (PBS, Life Technologies Europe B.V. The Netherlands) to remove the formalin [33]. Tensile testing was carried out in a Shimadzu Autograph ASG-X tensile machine with a 10-N load cell. A specific in-house fixture, consisting of two steel plates bolted together, was used to clamp each end of the samples, with sandpaper between the samples and the plates to prevent slipping (resulting in a total size of these 3D PC scaffolds of 1.5 × 1 cm). The gauge length of the implant samples was 10–12 mm and the width was 10 mm. With these settings, the tensile strength could be measured within the limits for normal bladder tissue (2.4 MPa) [24]. The crosshead speed was set to 2 mm/min. In total, 15 samples were analyzed, and five tests were performed at each time point (5, 10, and 28 days).

The elasticity of the implants was analyzed by post-processing the tensile test results; by these means, the elasticity refers to the stiffness of the implants during the initial elastic deformation. This parameter could enable the quantification of the resistance of the implants to deformation when certain loads were applied.

### 4.9. Statistical Analysis

Median and standard deviation of tensile strength and elasticity were calculated for every time point. Histological characterizations regarding epithelial and inflammatory markers were evaluated in at least two (out of four specimens) per time point. Means and standard deviations were assessed across all conditions, and the statistical difference was evaluated using a two-tailed, paired *t*-test with *p* < 0.05 considered as significant.

## 5. Conclusions

Autologous minced tissue placed within a hybrid construct of compressed collagen and knitted fabric can enable the expansion and reorganization of the epithelium in vivo without the need for in vitro cell culture. The method is simple and safe, and, in the future, it could be used as a one-stage procedure in any ordinary surgical setting for tissue expansion. The next step will be to evaluate the transplant with autologous bladder epithelium in a larger animal model, perhaps for bladder augmentation.

## Figures and Tables

**Table 1:** Rat identity, type of 3D scaffold, size, and time of retrieval. In total, nine rats were used for histological evaluation and, additionally, three were used for mechanical evaluation.

Rat ID	# 3D PC-Scaffolds	Size of the 3D Scaffold	Time to Termination after Transplantation	Assessment
II, V, VII	4 with minced skin 4 without minced skin	1 × 1 cm	5 days	Histology
IV	5 without minced skin	1.5 × 1 cm	5 days	Biomechanics
VIII, IX, X	4 with minced skin 4 without minced skin	1 × 1 cm	10 days	Histology
XII	5 without minced skin	1.5 × 1 cm	10 days	Biomechanics
I, III, VI	4 with minced skin 4 without minced skin	1 × 1 cm	4 weeks	Histology
XI	5 without minced skin	1.5 × 1 cm	4 weeks	Biomechanics

**Table 2:** Details of the antibodies and special staining methods used in this study.

Antibody/Stain	Target	Dilution Used
KI67 (Abcam ab16667)	For the identification of proliferative cells.	1:100
CD68 (Abcam ab31630)	For the identification of macrophages (type I).	1:100
Neutrophil Myeloperoxidase (Abcam ab9535)	For the identification of neutrophils.	1:100
MNF116 (DAKO M0821)	A broad-spectrum anti-keratin antibody reacting to cytokeratin 5, 6, 8, 17 and 19.	1:1000
Periodic Acid Schiff (PAS) Stain Kit (Mucin Stain) (ab150680)	Blood vessel walls and connective tissues are faintly stained with PAS reaction on the luminal surface (purple-magenta color). The lumen is visualized and erythrocytes stain turquoise.	According to manufacturer recommendations
Trichrome Stain Kit (Connective Tissue Stain) (ab150686)	Visualization of collagenous connective tissue fibers and red muscle fibers.	According to manufacturer recommendations

## Data Availability

Not applicable.

## Supplementary Materials

The following supporting information can be found at: https://www.mdpi.com/article/10.3390/ijms232012703/s1. Figure S1. Histological evaluation of transplants. Trichrome staining to illustrate the approach for assessment of vessel formation, inflammation, and proliferation. Each excised specimen was evaluated in toto, measuring the transplant in the area between the subcutaneous muscular level (star) to the native subcutaneous space. Visible remnants of the PCL mesh are marked (asterisk) as well as the entire transplant area (yellow line). Figure S2. Representative H&E staining of 3D PC constructs 5 (a–d), 10 (e–h), and 28 days (i–l) after in vivo transplantation under the rodent skin. Dashed circles illustrating infiltration of inflammatory cell into the collagen part of the construct (b, d, f, h). Asterisk showing residuals of the knitted PCL fabric (i, k), arrows showing vascularization (j, l). White triangle showing minced skin tissue on top of the 3D PC construct (c, g).
