# Supplementary figures and images for: Exploring the Concept of In Vivo Guided Tissue Engineering by a Single-Stage Surgical Procedure in a Rodent Model
Source: Int J Mol Sci. 2022 Oct 21;23(20):12703. doi: 10.3390/ijms232012703 (PMC9604108; doi:10.3390/ijms232012703)

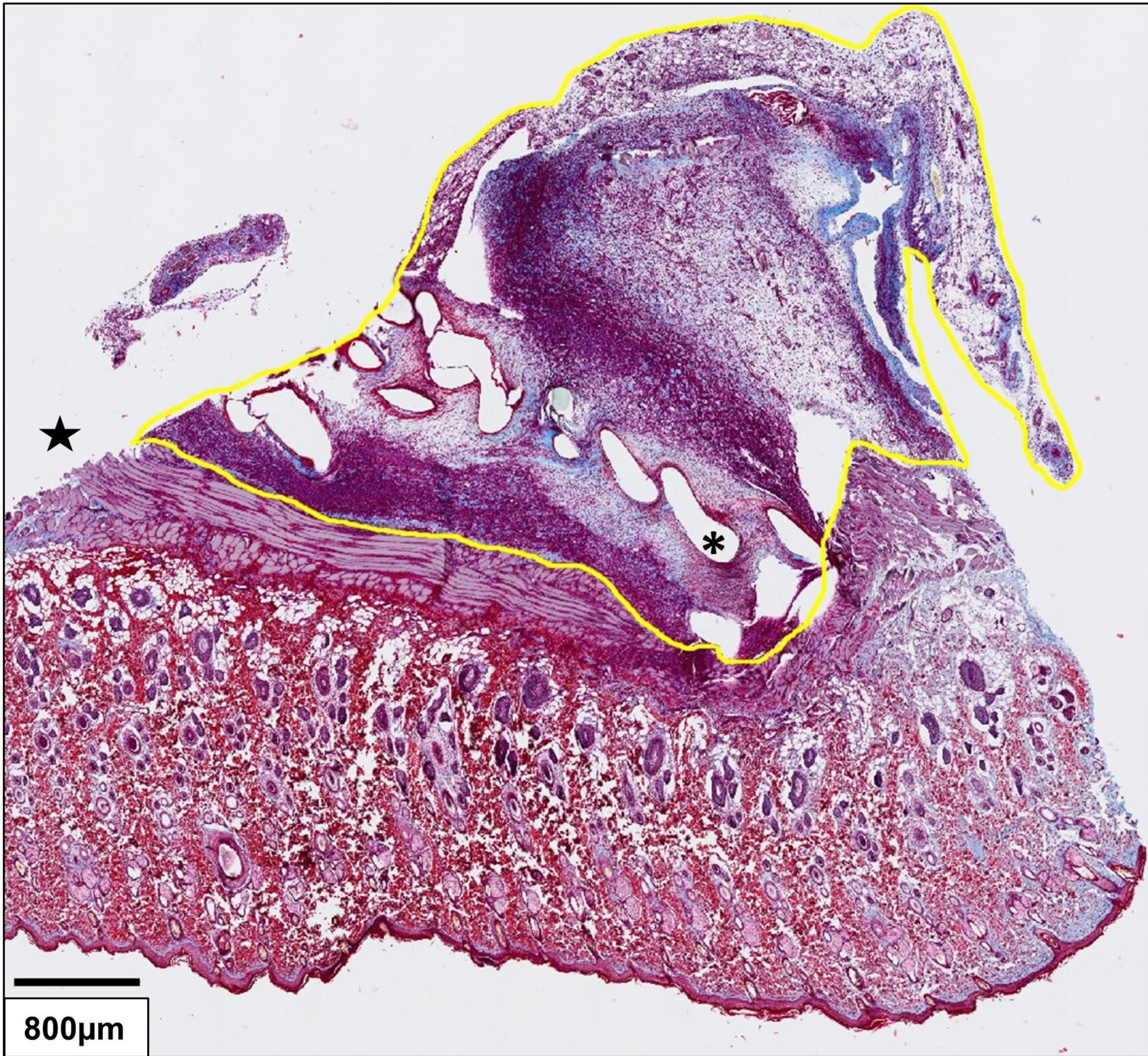

800μm

Supplement: Supplementary file 1 [file ijms-23-12703-s001.zip › Suplementary Figure S1.pdf]

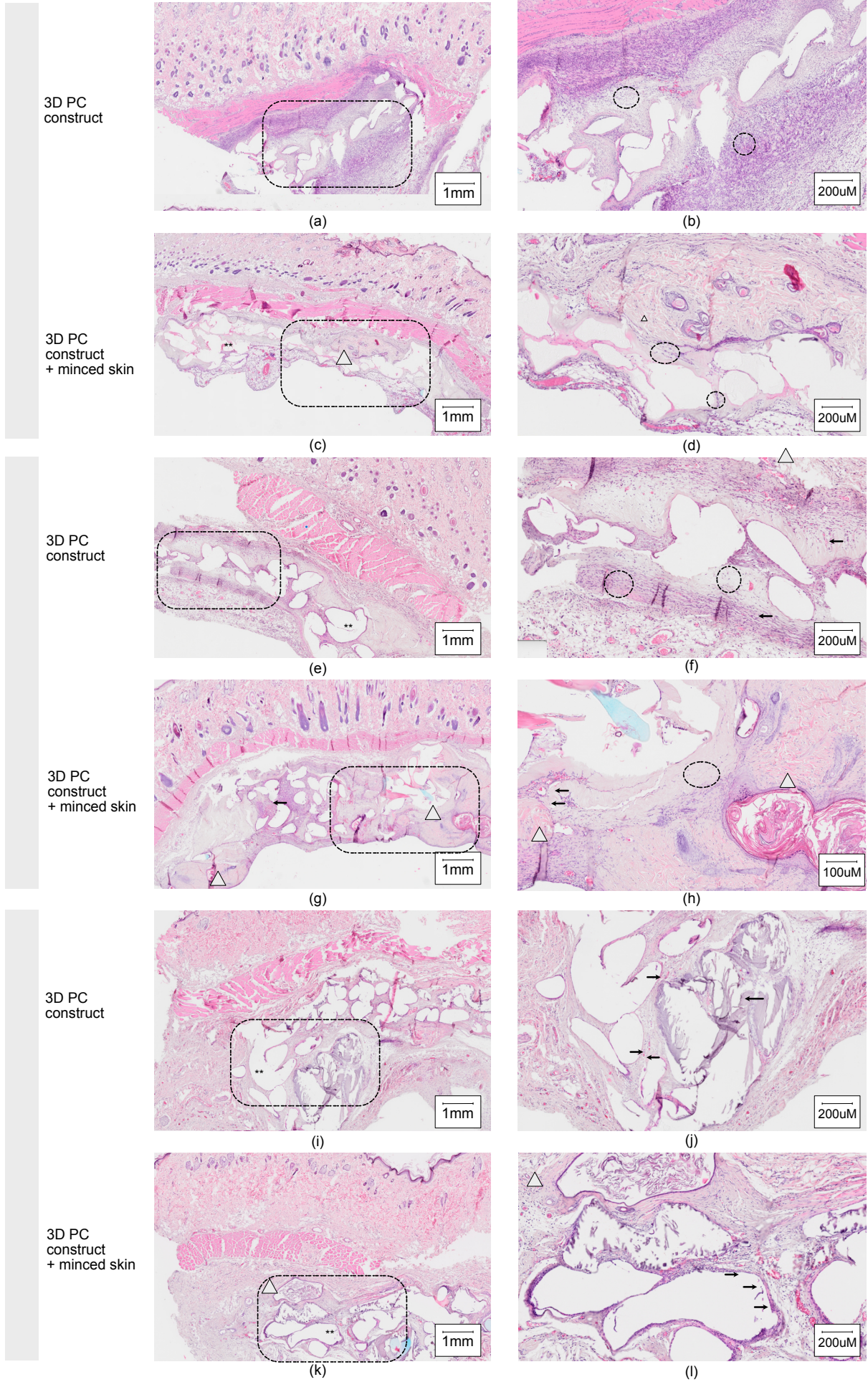

Supplement: Supplementary file 1 [file ijms-23-12703-s001.zip › Supplementary Figure S2.pdf]
